# Self-Reported Periodontitis and Incident Type 2 Diabetes among Male Workers from a 5-Year Follow-Up to MY Health Up Study

**DOI:** 10.1371/journal.pone.0153464

**Published:** 2016-04-26

**Authors:** Atsushi Miyawaki, Satoshi Toyokawa, Kazuo Inoue, Yuji Miyoshi, Yasuki Kobayashi

**Affiliations:** 1 Department of Public Health, Graduate School of Medicine, The University of Tokyo, Tokyo, Japan; 2 Department of Community Medicine, Chiba Medical Center, Teikyo University School of Medicine, Chiba, Japan; 3 Industrial physician, Meiji Yasuda Life Insurance Company, Tokyo, Japan; National Cardiovascular Center Hospital, JAPAN

## Abstract

**Aims:**

The purpose of this study was to examine whether periodontitis is associated with incident type 2 diabetes in a Japanese male worker cohort.

**Methods:**

The study participants were Japanese men, aged 36–55 years, without diabetes. Data were extracted from the MY Health Up study, consisting of self-administered questionnaire surveys at baseline and following annual health examinations for an insurance company in Japan. The oral health status of the participants was classified by two self-reported indicators: (1) gingival hemorrhage and (2) tooth loosening. Type 2 diabetes incidence was determined by self-reporting or blood test data. Modified Poisson regression approach was used to estimate the relative risks and the 95% confidence intervals of incident diabetes with periodontitis. Covariates included age, body mass index, family history of diabetes, hypertension, current smoking habits, alcohol use, dyslipidemia, and exercise habits.

**Results:**

Of the 2895 candidates identified at baseline in 2004, 2469 men were eligible for follow-up analysis, 133 of whom were diagnosed with diabetes during the 5-year follow-up period. Tooth loosening was associated with incident diabetes [adjusted relative risk = 1.73, 95% confidence interval = 1.14–2.64] after adjusting for other confounding factors. Gingival hemorrhage displayed a similar trend but was not significantly associated with incident diabetes [adjusted relative risk = 1.32, 95% confidence interval = 0.95–1.85].

**Conclusions:**

Tooth loosening is an independent predictor of incident type 2 diabetes in Japanese men.

## Introduction

Type 2 diabetes is a significant and growing health problem. As of 2013, approximately 382 million people worldwide have diabetes, and this number is predicted to reach 592 million by 2035 [1]. Type 2 diabetes is a chronic disease and requires long-term care. Additionally, the progression of complications leads to decreased quality of life and a high cost burden to the insurance system [2]. Therefore, prevention is vital. Recently, a number of lifestyle-related factors involved in type 2 diabetes development have been demonstrated, including obesity, poor diet, physical inactivity, smoking, and heavy alcohol use [3–6]. To prevent type 2 diabetes, it is useful to identify novel and influential risk factors that could easily be targeted for intervention. Recently, the relationship between type 2 diabetes and periodontitis has been identified [7]. However, the effects of periodontitis on incident type 2 diabetes have not yet been established, because many confounding factors and “reverse causation”; that is, poor glycemic control in diabetes patients may be a cause of periodontitis, exist between the two diseases [7].

Periodontal disease is a chronic infection caused primarily by plaque accumulation, including periodontitis bacterium in periodontal pockets. The disease is classified into two forms: gingivitis, in which inflammation is limited to the gum, and periodontitis, in which inflammation and tissue destruction occurs beyond the gum. Previous studies have demonstrated that people with periodontitis displayed elevated systemic inflammation levels [8]. Chronically elevated systemic inflammation leads to type 2 diabetes via insulin resistance [9,10]. Therefore, it is plausible that periodontitis leads to increased incident type 2 diabetes risk.

A previous study using the National Health and Nutrition Examination Survey samples found that moderate to severe periodontitis was associated with self-reported type 2 diabetes incidence [11]. However, this previous study did not include fasting plasma glucose (FPG) levels or HbA1c as criteria for type 2 diabetes diagnosis, which hampered the exclusion of people with undiagnosed diabetes at baseline. As a result, the effects of type 2 diabetes on periodontitis remained (a reverse causation). In a 7-year study using samples from civil service officers in Japan, moderate to severe periodontitis was significantly associated with increased incident type 2 diabetes risk using an unadjusted analysis, though significance was not detected using a multivariate analysis [12].

Thus, in this longitudinal study, we aimed to detect the effect of periodontitis on incident type 2 diabetes using self-administered questionnaires, with adjustment for possible confounding factors. We identified participants without diabetes at baseline using FPG levels or self-reporting to remove the reverse causation of diabetes on periodontal status at baseline.

## Materials and Methods

### Data collection

The study subjects were men aged 36 to 55 years that were employed by a large Japanese insurance company and had participated in the MY Health Up Study, a prospective study initiated in 2004 [13–15]. Self-administered questionnaires that asked about baseline health status (physical, mental, and oral) and lifestyle habits were distributed to all employees in October 2004. Of the 43064 questionnaires distributed, 34921 (81.1%) were collected. The applied code numbers were lost on two questionnaires, making it impossible to link the data. Health examinations (medical interview and routine check-up, including a blood test) were performed annually as an obligation of the employer.

**Fig 1:**
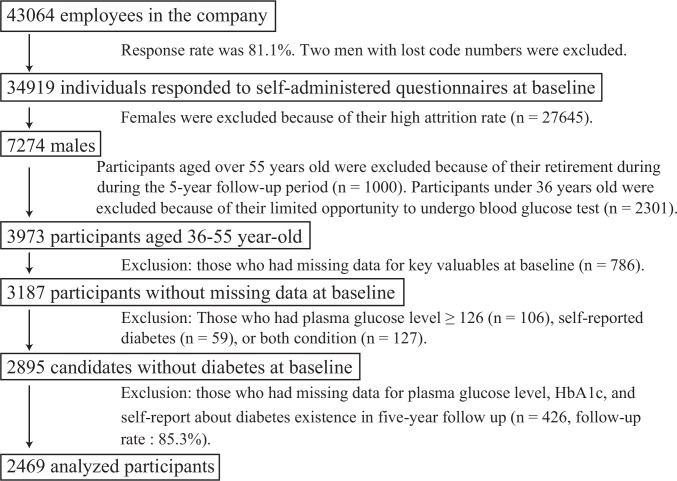
Identification of the 2469 participants.

In this study, the subjects were limited post hoc for analysis. Participant inclusion/exclusion criteria are displayed in Fig 1. We excluded women because most were part-time sales staff with a high turnover rate, making a 5-year follow-up assessment challenging. The remaining 7274 men were aged 19–83 years (42.1 years ± 10.8 years). At this company, most workers retire at age 60. The proportion of employees undergoing blood glucose test was quite low among men under 36 years old [27.4% (vs. 98.5% in men aged 36 years and older)] because the blood glucose test was not obligatory for young people in Japan. Therefore, we selected 3973 participants between 36 and 55 years of age. Among them, 786 participants who did not provide key data at baseline were excluded. An additional 292 participants with diabetes at baseline were excluded. Diabetes at baseline was identified by either self-reporting or FPG level ≥ 126 mg/dl. We did not use the data of oral glucose tolerance test (OGTT) and HbA1c at baseline because those were not measured. Thus, the candidates for analyses comprised of 2895 men without diabetes at baseline. Among these candidates, we analyzed 2469 participants without missing values in the 5-year health examination records (follow-up rate: 85.3%). The Institutional Review Board of The University of Tokyo approved this study protocol after ethical consideration (Approval No. 1021). The researchers of this study have no conflicts of interest to declare. This study is an observational study. We informed all the employees in this company about the purpose and methods of this study in a written form when the baseline self-administered questionnaires were distributed. Only the employees who consented to participate in this study responded to the questionnaires. These participants had the option to opt out from the study.

### Periodontal status and covariates at baseline

In the current study, we chose self-reported periodontal symptoms as indicators of periodontal status. In a previous study discussing validity of self-reported periodontal health, gingival hemorrhage was relatively sensitive for severe periodontitis (maximum attachment loss of ≥ 7 mm was used as a criterion of periodontitis), while tooth loosening was specific for severe periodontitis (maximum attachment loss of ≥ 7 mm) [16]. In another study, gingival hemorrhage was relatively specific for bleeding on probing, which suggests Community Periodontal Index (CPI) code ≥ 1 [17]. At baseline, participants were asked the following two questions regarding their periodontal status: “Do you have gingival bleeding?” and “Do you have tooth loosening?” Based on the answers to these questions, participants’ periodontal status (the primary independent variable) was classified into two indicators; (1) gingival hemorrhage and (2) tooth loosening.

We included age, body mass index (BMI), family history of diabetes, hypertension, current smoking habits, alcohol use, dyslipidemia, exercise habits, and prediabetes as covariates. Recent studies have suggested that reduced physical activity is associated with type 2 diabetes [18]. We considered family history of diabetes to be a surrogate marker for genetic factors. Prediabetes leads to type 2 diabetes [19]. Furthermore, prediabetes was associated with severe periodontitis in a recent cross-sectional study [20,21]. Periodontitis causes insulin resistance via systemic inflammation [7,8,10], while prediabetes may promote compositional shifts in the subgingival microbiome and contribute to gingival inflammation [21]. Prediabetes may also be a confounding factor as well as an intervening factor in the causal effect of periodontitis on incident type 2 diabetes. Therefore, we additionally controlled for prediabetes at baseline as a sensitivity analysis.

BMI, family history of diabetes, and heavy alcohol consumption (≥ 40 g/day) at baseline were determined from the medical check-up or interview in 2004. We defined heavy alcohol consumption as consuming more than 40 g/day [22]. BMI was calculated by dividing weight (kg) by height squared (m^2^). A medical history of hypertension (current or past), current smoking habits, and exercise habits (more than 30 minutes of exercise twice or more per week) were derived from the baseline self-administered questionnaire. Hypertension was identified not only from the self-administered questionnaire, but also from raw data [systolic blood pressure ≥ 140 mmHg or diastolic blood pressure ≥ 90 mmHg] or anti-hypertensive drug usage in medical check-up in 2004 [23]. Dyslipidemia at baseline was diagnosed by blood test in 2004 [triglycerides ≥ 150 mg/dl, high-density lipoprotein ≤ 40 mg/dl, or low-density lipoprotein ≥ 140 mg/dl, calculated according to the Friedewald equation for total cholesterol, high-density lipoprotein, and triglyceride levels] [24]. We used the term “prediabetes” to reflect impaired fasting glycaemia (IFG), which was defined as an FPG level ranging from 100 mg/dl to 125 mg/dl, according to the American Diabetes Association criteria and the Japan Diabetes Society’s committee criteria [25,26].

### Incident type 2 diabetes

Type 2 diabetes was diagnosed if a person without diabetes at baseline self-reported “diabetes” or met blood test criteria at least once during the 5-year follow-up period. We assumed that those who take diabetes medication reported his diabetes by themselves. The blood test criteria were FPG level ≥ 126 mg/dl between 2005 and 2009 and/or HbA1c values ≥ 6.5% in 2008 and 2009 [25]. In our data, HbA1c from 2005 to 2007 and OGTT during all the study period were unavailable because they were not obligatory during respective periods. All blood tests were conducted in the morning before breakfast.

### Statistical analysis

The characteristics of the participants with and without gingival hemorrhage and tooth loosening were compared. The t-test and chi-square test were performed for each of the covariates and outcome. Because the outcome of this study, incident type 2 diabetes, is not necessarily a rare event (later shown). Modified Poisson regression approach (Poisson regression with robust standard errors) was used to calculate adjusted relative risks (RRs) and 95% confidence intervals (CIs) of incident type 2 diabetes with periodontitis [27]. Two indicators based on self-reported periodontal status were used as independent factors associated with periodontitis: gingival hemorrhage (Model 1) and tooth loosening (Model 2). Covariates were age, BMI, genetic factors (family history of diabetes), health behaviors (current smoking habits, alcohol use, and exercise habits), vascular risk factors (hypertension and dyslipidemia), and prediabetes. Prediabetes had not been previously established as a covariate. Therefore, to examine the influence on the relationship between periodontitis and diabetes, we first analyzed excluding prediabetes as a covariate and then analyzed including prediabetes as a covariate. Although we excluded women due to their high turn-over rate as mentioned above, in a complementary analysis, we analyzed the women aged 36–55 years who were excluded from the original population in the same way as the main analysis. Statistical analyses were performed using R version 3.1.3 and Stata 13 for Windows. P values < 0.05 were considered statistically significant.

## Results

Of the 2895 men without diabetes at baseline, 2469 were eligible for follow-up analysis, resulting in a follow-up rate of 85.3%. Of the 2469 study participants, 133 were newly diagnosed with type 2 diabetes during the 5-year period (113 diagnosed only by blood test, one identified only by self-report, and 19 identified by both criteria). The type 2 diabetes incidence rate was 11.2 per 1000 person-years.

Table 1 displays the baseline characteristics of the participants. In comparisons to variables at baseline, age, BMI, and the hypertension prevalence were more likely to increase in participants with each periodontal symptom. Compared with counterparts, the percentage of smokers was significantly lower among people with self-reported gingival hemorrhage, while significantly higher in people with self-reported tooth loosening. This is consistent to the fact that smoking has a suppressive effect on gingival hemorrhage, but is a risk factor for periodontitis [28,29]. The rate of incident diabetes seems to increase in people with each periodontal symptom.

**Table 1:** Baseline characteristics of the 2469 participants.

	Gingival hemorrhage			Tooth loosening		
	yes (n = 795)	no (n = 1674)	*P* value	yes (n = 262)	no (n = 2207)	*P* value
Age, mean (SD)	45.1 (5.80)	44.7 (5.98)	0.059	48.1 (5.40)	44.4 (5.87)	<0.001
BMI, mean (SD)	24.3 (2.91)	24.0 (3.00)	0.044	24.6 (2.93)	24.0 (2.97)	0.002
Family history of diabetes, n (%)	103 (13.0)	189 (11.3)	0.231	29 (11.1)	263 (11.9)	0.688
Hypertension, n (%)	210 (26.4)	377 (22.5)	0.034	75 (28.6)	512 (23.2)	0.051
Current smoking, n (%)	329 (41.4)	798 (47.7)	0.003	158 (60.3)	969 (43.9)	< 0.001
Alcohol use (≥ 40g/day), n (%)	157 (19.7)	302 (18.0)	0.308	67 (25.6)	392 (17.8)	0.002
Dyslipidemia, n (%)	361 (45.4)	770 (46.0)	0.784	134 (51.1)	997 (45.2)	0.067
Exercise habits, n (%)	71 (8.9)	190 (11.4)	0.068	26 (9.9)	235 (10.6)	0.718
Prediabetes, n (%)	232 (29.2)	435 (26.0)	0.095	92 (35.1)	575 (26.1)	0.002
Incident diabetes, n (%)	53 (6.7)	80 (4.8)	0.052	29 (11.1)	104 (4.7)	< 0.001

BMI: body mass index; Prediabetes: fasting plasma glucose 5.6 mmol/l (100mg/dl) to 6.9 mmol/l (125mg/dl); Exercise habits: >30 minutes/day and ≥2days/week. Age and BMI were tested by the t-test, and the other factors were tested by the Pearson’s chi-square test.

Baseline oral status by age group is displayed in Table 2. The number of participants who reported tooth loosening tended to increase with age, while gingival hemorrhage was not impacted by age group. In every age group, there were fewer participants who reported tooth loosening than those who reported gingival hemorrhage.

**Table 2:** The baseline self-reported oral status by age group.

	Gingival hemorrhage	Tooth loosening
Age (n)	n (%)	n (%)
36‒40 years old (769)	223 (29.0)	37 (4.8)
41‒45 years old (599)	200 (33.4)	35 (5.8)
46‒50 years old (530)	185 (34.9)	84 (15.8)
51‒55 years old (571)	187 (32.7)	106 (18.6)
all (2469)	795 (32.2)	262 (10.6)

The number and percentage of participants who answered “yes” to the following two questions are shown for every age-group; “Do you have gingival bleeding?” and “Do you have tooth loosening?”

Table 3 shows the results of modified Poisson regression analysis performed using the two models described in the Patients and Methods section. In Model 2, tooth loosening was associated with incident type 2 diabetes [adjusted RR = 1.73, 95% CI = 1.14–2.64]. In Model 1, gingival hemorrhage was not significantly associated [adjusted RR = 1.32, 95% CI = 0.95–1.85]. After controlling for prediabetes, the association between tooth loosening and incident type 2 diabetes was still evident [adjusted RR = 1.73, 95% CI = 1.18–2.53], while gingival bleeding was still not associated [adjusted RR = 1.23, 95% CI = 0.90–1.70]. In each modified Poisson regression model, significant associations with incident type 2 diabetes were evident for BMI, family history of diabetes, hypertension history, and prediabetes, but were not identified for current smoking habits, drinking habits, dyslipidemia, or exercise habits.

**Table 3:** The influence of the periodontal status on incident type 2 diabetes in modified Poisson regression analysis.

	Adjusted Relative Risk for incident diabetes (95% confidence interval)			
	Model 1		Model 2	
Independent Variables	A	B	A	B
Prediabetes	‒	5.89 (4.06 ‒ 8.56)‡	‒	5.94 (4.08 ‒ 8.63)‡
(reference: no prediabetes)				
Gingival hemorrhage	1.32 (0.95 ‒ 1.85)	1.23 (0.90 ‒ 1.70)	‒	‒
(reference: no gingival hemorrhage)				
Tooth loosening	‒	‒	1.73 (1.14 ‒ 2.64)*	1.73 (1.18 ‒ 2.53)†
(reference: no tooth loosening)				

*: P<0.05

†: P<0.01

‡: P<0.001.

BMI: body mass index. Model 1 used gingival hemorrhage as an oral status indicator. Model 2 used tooth loosening as an oral status indicator. A: not including prediabetes as a covariate. B: including prediabetes as a covariate. The other covariate consisted of age, current smoking habits, BMI, family history of diabetes, hypertension, alcohol heavy consumption (≥ 40 g/day), and exercise habits (> 30 minutes, ≥ 2 days/week).

In a complementary analysis conducted for women aged 36–55 years, neither gingival hemorrhage nor tooth loosening impacted on incident diabetes significantly [S1 Table: adjusted RR = 0.99, 95% CI = 0.79–1.08; adjusted RR = 1.08, 95% CI = 0.81–1.43, respectively].

## Discussion

In this 5-year follow-up study, we demonstrated that self-reported tooth loosening was a significant predictor of incident type 2 diabetes among middle-aged men. The increased risk was independent of other risk factors, including age, BMI, family history of diabetes, hypertension, current smoking habits, alcohol use, dyslipidemia, and exercise habits. Even after adjusting for the influence of prediabetic state on incident type 2 diabetes to consider a causal bidirectional relationship, this association remained significant. Tooth loosening reflects severe periodontitis [16,30,31]. Thus, our results suggest that severe periodontitis is associated with type 2 diabetes incidence.

Accordingly, self-reported tooth loosening may be a useful piece of information to identify people at an increased risk of developing type 2 diabetes. In contrast, gingival hemorrhage displayed a similar trend, but we did not identify any statistically significant association in our study. In the group with self-reported tooth loosening, the proportion of people with advanced periodontitis leading to eventual tooth loss might be higher than in the group with self-reported gingival hemorrhage [16,30].

To date, there have only been a few longitudinal studies investigating periodontitis as a cause for type 2 diabetes. Using National Health and Nutrition Examination Survey data, Demmer et al. demonstrated that participants with intermediate-level periodontitis displayed a twofold increased risk of incident type 2 diabetes compared with that of participants with a healthy periodontal status [11]. Demmer’s work is the first population-based longitudinal study on this topic but is limited in that diabetes was identified by self-reporting. In a Japanese cohort study, Ide et al. could not confirm the effects of moderate or severe periodontitis on incident diabetes after adjusting for possible covariates [12]. Notably, the SHIP study by Demmer et al. revealed that the participants with severe periodontitis presented with an approximate fivefold increase (about 0.1%) in HbA1c over five years compared with that of individuals without periodontitis [32]. However, these previous studies did not consider prediabetic state at baseline. Prediabetes leads to incident type 2 diabetes [19] and may be correlated with severe periodontitis [20,21]. Thus, prediabetes may be a confounding factor in the association between periodontitis and incident type 2 diabetes. In this study, though prediabetes prevalence was higher among people with tooth loosening, we demonstrated that tooth loosening was associated with incident type 2 diabetes, even after adjusting for prediabetic state.

The biological mechanism of the association between periodontitis and type 2 diabetes has not been sufficiently clarified, though it is thought that systemic inflammation caused by periodontitis may lead to type 2 diabetes. Serum IL-6 and C-reactive protein levels are high in people with periodontitis [8]. Inflammatory markers have been suggested to increase the risk of incident type 2 diabetes [9]. Elevated C-reactive protein (CRP) levels are also related to insulin resistance [10]. In addition, recent studies have indicated that adipokines may impact both periodontitis and type 2 diabetes susceptibility [7].

We added analyses of elevated CRP level (> 0.3 mg/dl) and high white blood cell count (> 1.0×10^4^/μl) at baseline as covariates. They were not significantly associated with incident type 2 diabetes. As CRP and white blood cell count are acute inflammation markers with relatively short half-life, utilizing these markers may not be relevant as an indicator of chronic inflammation.

The systemic inflammatory pathways common in both periodontitis and type 2 diabetes may indicate a positive linear association between severe periodontitis and incident type 2 diabetes. Tooth loosening reflects a severe periodontal status. On the other hand, gingival hemorrhage reflects mild periodontal disease as well as severe periodontitis [30,31]. This may explain why only tooth loosening was significantly associated with incident type 2 diabetes in this study. The tooth loosening or gingival hemorrhage RR did not change remarkably after controlling for prediabetes. This may be due to endogeneity of prediabetes for other covariates, but this topic is beyond the scope of our study.

In this study, we excluded young male employees as they have limited opportunity to undergo blood glucose test. However, to allow robustness, we performed analysis in the same way without excluding male under 36 years old, but the result was almost the same. In contrast, in complementary analysis for women aged 36–55 years, we did not detect any clear association between self-reported periodontitis and incident type 2 diabetes. In this insurance company, vast majority of the sales staffs were female. They were non-regular employees and tended to show high turn-over rate [44.6% (vs. 85.3% in men)]. In general, it is known that health attitude and health status of non-regular workers are worse than those of regular workers [33]. Therefore, female subjects for analysis might be limited to relatively healthy population and the impact might be substantially distorted. Additionally, the other factors such as pregnancy and menopause might confound the relationship.

Some limitations exist in this study. First, we did not include HbA1c at baseline or OGTT in follow-up period due to data limitation. This may lead to underestimation of the number of people with diabetes, though the type 2 diabetes incidence rate in this study was 11.2 per 1000 person-years, similar to a previous study in Japanese middle-aged men [6]. Second, we assessed oral status by self-administered questionnaire responses at baseline. Self-reported gingival hemorrhage and tooth loosening were moderately sensitive and specific [16,17], though self-reporting may lead to misclassification, which could have weakened the association between periodontitis and type 2 diabetes. This would be unlikely, however, to lead to a false positive result. In addition, some covariates were measured based on the same self-administered questionnaires. This might cause correlation among measurement errors of exposures and covariates. This might potentially distort the result to an unknown degree. Third, oral status during follow-up could change after a dentist visit or periodontitis progression, leading to potential misclassification, which could have weakened the targeted association. Fourth, confounding factors of the relation between periodontitis and type 2 diabetes might not be sufficiently adjusted in this study. A relationship between socioeconomic status and diabetes has been suggested [34]. However, we did not include socioeconomic status among the covariates, because the subjects were men aged 36–55 years employed at a large Japanese insurance company, thus belonging to a relatively homogenous population. In addition, healthy attitudes are likely to influence both diabetes and periodontitis. We controlled for alcohol use, exercise habits, and current smoking habits as covariates related to health attitudes, though other confounding factors such as diet might exist. Finally, the subjects for analysis in this study were middle-aged male employees who answered baseline questionnaires about health status, and could undergo health examinations every year for five years. Thus, as a group, the finally analyzed subset might potentially be healthier than the group with missing variables. This selection bias might distort the result and needs to be addressed. Among the 3973 male participants aged 36–55 years, those who had missing key variables at baseline or during the follow-up period did not show remarkable differences in age, BMI, FPG, systolic/diastolic blood pressure, and the prevalence of dyslipidemia at baseline, compared with those without missing variables [mean (SD): 45.2 (6.8) vs. 45.1 (6.0), P value = 0.658, 24.1 (3.4) vs. 24.3 (3.1), P value = 0.06, 101.5 (24.1) vs. 100.1 (30.9), P value = 0.143, 117.3 (17.0) vs. 117.0 (16.3), P value = 0.678, 71.6 (11.7) vs. 71.7 (11.2), P value = 0.903, 48.5% vs. 46.5%, P value = 0.347, respectively]. However, concerns remain that the post hoc subject restriction might potentially raise the selection bias.

This study suggests that severe periodontitis impacts incident type 2 diabetes among middle-aged men. Among Japanese middle-aged people in general, over 80% have some gingival abnormality (CPI ≥ 1), and one third have moderate to severe periodontitis (periodontal pocket depth ≥ 4 mm) [35]. Periodontitis treatment decreases systemic inflammation levels and may improve insulin resistance [36]. The possibility that periodontal treatment affects glycemic control in people with type 2 diabetes has been previously reported [37]. Thus, a population approach to prevent progression to severe periodontitis or to identify high-risk individuals with severe periodontitis may be useful for incident type 2 diabetes prevention. Self-reported tooth loosening is potentially a non-invasive, affordable, and easy indicator of a high risk of diabetes.

The number of people with type 2 diabetes is rapidly increasing, not only in industrialized countries like Japan, but also in low- to middle-income countries, and low-cost preventative measures are required [1]. Tooth brushing, dental floss use, and tobacco cessation are relatively simple and economical ways to prevent severe periodontitis [30]. According to our findings, such interventions in people with periodontitis may lead to a decrease in type 2 diabetes risk.

The effect of mild periodontal disease on incident type 2 diabetes is uncertain. Therefore, it also remains unclear whether treatment of mild periodontitis would prevent incident type 2 diabetes. In this study, gingival hemorrhage, which contains mild periodontal disease, showed a similar trend with tooth loosening (no significance). Precise measurement of periodontal status and longer follow-up might reveal an association of mild periodontal disease with incident type 2 diabetes. Future studies are warranted.

## Conclusion

In summary, we have demonstrated that self-reported tooth loosening is an independent predictor for incident type 2 diabetes in Japanese male workers. The relationship between oral health and diabetes has drawn attention recently, but the utilization of oral health information in general medical practice is not necessarily sufficient because the assessment of oral health by professionals is time consuming. In this perspective, self-reported tooth loosening is an effective indicator that suggests necessity of further medical examination.

## Supporting Information

S1 Table The influence of oral health indicators on incident type 2 diabetes in modified Poisson regression analysis for women. (PDF)
