# Supplementary material for: Self-Reported Periodontitis and Incident Type 2 Diabetes among Male Workers from a 5-Year Follow-Up to MY Health Up Study
Source: PLoS One. 2016 Apr 26;11(4):e0153464. doi: 10.1371/journal.pone.0153464 (PMC4846029; doi:10.1371/journal.pone.0153464)
Supplement: S1 Table — (PDF) [file pone.0153464.s001.pdf]

S1 Table. The influence of the oral health indicators on incident type 2 diabetes in modified Poisson regression analysis for women.

Independent Variables	Adjusted Relative Risk for incident diabetes（95%confidence interval) for women aged 36-55 years.	
	Model 1	Model 2
Gingival hemorrhage (reference: no gingival hemorrhage)	0.99 (0.79–1.24)	-
Tooth loosening (reference: no tooth loosening)	-	1.08 (0.81–1.43)

Model 1 used gingival hemorrhage as an oral status indicator. Model 2 used tooth loosening as an oral status indicator.
The other covariates consisted of age, current smoking habits, BMI, family history of diabetes, hypertension, alcohole heavy consumption, and exercise habits.
